# Maize varietal replacement in Eastern and Southern Africa: Bottlenecks, drivers and strategies for improvement

**DOI:** 10.1016/j.gfs.2021.100589

**Published:** 2022-03

**Authors:** Walter Chivasa, Mosisa Worku, Adefris Teklewold, Peter Setimela, James Gethi, Cosmos Magorokosho, Nicholas J. Davis, Boddupalli M. Prasanna

**Affiliations:** a International Maize and Wheat Improvement Center (CIMMYT), ICRAF Campus, UN Avenue, Gigiri, P.O. Box 1041–00621, Nairobi, Kenya; b CIMMYT, Addis Ababa, Ethiopia; c CIMMYT, P.O. Box MP163, Harare, Zimbabwe

**Keywords:** PLC, Product Lifecycle Management, Maize, Varietal replacement, Sub-Saharan Africa, Genetic gain, Product life cycle management

## Abstract

Seed security is vital for food security. Rapid-cycle, climate-adaptive breeding programs and seed systems that deliver new, elite varieties to farmers to replace obsolete ones can greatly improve the productivity of maize-based cropping systems in sub-Saharan Africa (SSA). Despite the importance and benefits of accelerated varietal turnover to climate change adaptation and food security, the rate of maize varietal replacement in SSA is slow. This review outlines the major bottlenecks, drivers, risks, and benefits of active replacement of maize varieties in eastern and southern Africa (ESA) and highlights strategies that are critical to varietal turnover. Although there is an upsurge of new seed companies in ESA and introduction of new varieties with better genetics in the market, some established seed companies continue to sell old (over 15-year-old) varieties. Several recently developed maize hybrids in ESA have shown significant genetic gains under farmers’ conditions. Empirical evidence also shows that timely replacement of old products results in better business success as it helps seed companies maintain or improve market share and brand relevance. Therefore, proactive management of product life cycles by seed companies benefits both the farmers and businesses alike, contributing to improved food security and adaptation to the changing climate.

## Highlights

• Maize varietal replacement is slow in many countries in sub-Saharan Africa, despite the growth in recent years of the region’s maize seed industry.
• Continued use of old, obsolete varieties exposes farmers to risks due to changing climate and emergent diseases and pests.
• Varietal replacement is required to translate genetic gains to on-farm productivity and help farmers adapt to climate change.
• Proactive product life cycle management improves seed business success and enhances market share and brand relevance.

## 1 Introduction

Maize-based cropping systems in Africa are affected by multiple stresses due to climate variability (Prasanna et al., 2021a), exposing farmers to significant risks to their food security and livelihoods, and severely testing their adaptation capacity (Adger et al., 2007). These cropping systems require not only improved varieties with tolerance to multiple stresses, but also active varietal turnover to mitigate the negative effects of the changing climate (Atlin et al., 2017). “Varietal turnover” is defined as the “replacement by farmers of an older variety with a more recently developed improved variety, a process that entails a genetic change” (Spielman and Smale, 2017). However, in sub-Saharan Africa (SSA), varietal turnover^1^ in general is exceptionally slow (Atlin et al., 2017). Farmers are still growing maize varieties that are more than 20 years old (Abate et al., 2017), which are not well-equipped to tackle the changing climates and emergence of new threats, including diseases and pests.

In a dynamic seed industry, increased genetic gains and better capacity to adapt to the changing climate and multiple stresses are realized through shorter breeding cycles (e.g., by using doubled haploidy and genomics-assisted breeding), supported by seed systems that quickly replace older varieties with new, improved genetics through active product life cycle (PLC) management. Maize yields in the USA, China, and Brazil have increased three-fold since early 1960s, driven by development of single-cross hybrids, shorter breeding cycles, competitive seed industries, and rapid varietal turnover (Atlin et al., 2017). However, in SSA, maize yields have made modest improvement over the decades with significant heterogeneity within the continent (Cairns et al., 2013; Atlin et al., 2017), despite significant genetic gains reported by international maize breeding programs of the CGIAR (Consultative Group on International Agricultural Research), through trials done on-station and on-farm (Badu-Apraku et al., 2013a, Badu-Apraku et al., 2013b, Badu-Apraku et al., 2015; Masuka et al., 2017a, b; Setimela et al., 2017, 2018).

Significant genetic gains in the CGIAR maize breeding programs in SSA are attributed to effective climate-adaptive breeding systems, robust phenotyping capacity, and extensive germplasm testing networks (Cairns et al., 2013; Masuka et al., 2017a, b; Prasanna et al., 2021a). The phenotyping system mimics the future climates by exposing breeding materials to controlled biotic and abiotic stresses (e.g., pests and diseases, managed drought, low nitrogen, etc.) to facilitate selection for relevant traits that, according to climate projections, are expected to be critical performance factors in the target population of environments (Prasanna et al., 2021a). This system has resulted in successful development and release of multiple-stress tolerant varieties, with yield advantages of up to 25% under stress conditions over the market-dominant commercial varieties in eastern and southern Africa (ESA) (Setimela et al., 2017, 2018). For example, CGIAR-derived maize hybrids have achieved genetic yield gains ranging from 21 to 141 kg ha^−1^ yr^−1^ under multiple stress environments in ESA (Masuka et al., 2017a, b), and up to 40 kg ha^−1^ yr^−1^ under multiple stress conditions in West Africa (Badu-Apraku et al., 2015). However, except for a few countries like Ethiopia (Abate et al., 2015; Ertiro et al., 2019), Rwanda (AGRA, 2017) and Uganda, these genetic gains have not effectively translated into increased grain yields on smallholder farms, due to various factors, especially slow varietal turnover, and poor agronomic practices. Faster varietal turnover combined with good agronomic practices in farmers’ ﬁelds improve yield and adaptation to climate change (Ertiro et al., 2019), leading to improved food security (Lunduka et al., 2018; Cairns and Prasanna, 2018).

Efficient breeding and seed systems that effectively deliver improved varieties to farmers have three major elements (Atlin et al., 2017): rapid breeding cycles, effective selection, and rapid varietal turnover. Reducing breeding cycles alone may not lead to climate change adaptation unless the new products replace the old/obsolete ones in the farmers' ﬁelds (Atlin et al., 2017). The rate of breeding should be in synchrony with seed delivery systems and replacement rates (Challinor et al., 2016). AGRA's Program for Africa's Seed Systems (PASS) was set up to address key bottlenecks in African seed sector, including training of plant breeders, development of new crop varieties, creation and/or support to new private seed companies, and establishment of agro-dealer networks (AGRA, 2017). This has led to the formation of more than 100 new seed companies in ESA, and release of several new crop varieties with better genetics. However, a lot still remains to be done on the varietal turnover front, as can be seen in case of maize (Table 1).

**Table 1:** Average age and market share of improved maize varieties presently grown in selected countries in the ESA.

Age (years)	Ethiopia		Uganda		Tanzania		Malawi^a^		Mozambique		Zambia		Zimbabwe	
	Volume (MT)	Share (%)	Volume (MT)	Share (%)	Volume (MT)	Share (%)	Volume (MT)	Share (%)	Volume (MT)	Share (%)	Volume (MT)	Share (%)	Volume (MT)	Share (%)
<10	26,440	67.6	9,298	73.8	3,192	48.6	5,905	28.2	2553	92.2	5,415	43.1	10,911	37.3
10–14	5,744	14.7	3,309	26.2	56	0.9	8,977	42.9	–	–	1,659	13.2	11,731	40.1
≥15	6,925	17.7	–	–	3,315	50.5	6,067	28.9	217	7.8	5,486	43.7	6,598	22.6
**Total**	**39,109**	**100**	**12,607**	**100**	**6,563**	**100**	**20,949**	**100**	**2770**	**100**	**12,560**	**100**	**29,239**	**100**

a Based on 2021 certified seed production figures; for other countries the data used was from 2020.

The current state of knowledge on the bottlenecks, risks and drivers for maize varietal replacement in ESA is inadequate. The objective of this review is to highlight the bottlenecks affecting varietal replacement, benefits and risks of rapid varietal turnover, and strategies to accelerate varietal turnover given that the area-weighted average age of maize varieties grown in some sub-regions/countries in SSA is still high (Krishna et al., 2021).

## 2 Area-weighted average age of maize varieties grown in SSA

Abate et al. (2017) found the area-weighted average age (AWAA) of maize varieties to be 14, 15, and 16 years in Eastern, Western, and Southern African markets, respectively. The estimated AWAA was 13 and 18 years for hybrids and open-pollinated varieties (OPVs), respectively, and 15 years across hybrids and OPVs. Walker (2015) estimated the AWAA of maize varieties in SSA to be 13 years. It takes on average seven to ten years to develop a maize variety through breeding in SSA, before the product is first registered for commercial production and sale. Thus, the current varieties grown by the farmers are much older than reported because the age of the varieties is often based on the year of official registration. It takes an additional two to three years from varietal release to seed scale-up and promotion in ESA (Mabaya et al., 2021). International Maize and Wheat Improvement Center (CIMMYT)-related improved maize varieties in ESA have shown a steady decrease in the overall AWAA from 14 years in 2014 to 10 years in 2021 (Table 2). Such a progress in maize varietal turnover in ESA could be attributed to the strengthening of seed systems including release of better genetics and intensive deployment through public-private partnerships.

**Table 2:** The area-weighted average age of CIMMYT-related improved maize varieties in the ESA in 2021 (based on 2020/2021 certified seed production and commercialization data).

Country	Certified seed production (tons) in 2020–2021	Estimated area (in ha) in 2021	Area-weighted Average Age (in years)
Ethiopia	38,386	1,744,802	11.71
Kenya	9,908	450,384	10.07
Tanzania	6,523	296,513	11.81
Uganda	12,607	573,050	7.69
**Eastern Africa**	**67,424**	**3,064,749**	**10.32**
Malawi	4,709	208,924	10.09
Mozambique	2,590	100,364	8.08
Zambia	12,684	576,542	10.40
Zimbabwe	22,145	956,627	10.56
**Southern Africa**	**42,127**	**1,842,458**	**9.78**
**Overall-ESA**	**109,552**	**4,907,207**	**10.05**

## 3 Active varietal turnover: benefits, bottlenecks, and drivers

### 3.1 Benefits of active varietal replacement

The advantages of varietal replacement are known. The genetic gains in grain yield can only translate into farmer productivity if improved varieties are rapidly disseminated and old ones replaced (Veettil et al., 2018). Regular varietal replacement improves productivity, averts potential yield losses due to devastating insect-pests and diseases (Witcombe et al., 2016; Prasanna et al., 2020, 2021b), climate change (Ray et al., 2012), and prevents loss of market share for seed companies. Rapid varietal replacement benefits farmers and seed companies and improves national food security, especially as climate change accelerates. The effect of climate change on crop pest and disease epidemics were well-documented (e.g., Garrett et al., 2006). Climate change predictions have shown an increased rainfall in eastern Africa and a decrease in southern Africa (IPCC, 2014). Increased precipitation may produce temporal overlap of seasons providing a ‘green bridge’ conducive for insect vectors like leafhoppers (*Cicadulina* species) that transmit maize streak virus (MSV) to survive throughout the year. Conversely, increased droughts followed by erratic rainfall at the beginning of the crop season have been associated with MSV epidemics in West Africa in 1983 and 1984 (Rosell and Thottappilly, 1985) and East Africa in 1988–1989 (Njuguna et al., 1990).

The damaging effects of a slow varietal turnover are nontrivial for farmers and seed companies alike. Slow varietal replacement leads to yield stagnation or decline, poor adaptation to climate change (Ray et al., 2012), and increases the vulnerability of farmers to risks associated with pest and disease outbreaks. Continuing with old, obsolete varieties also leads to customer dissatisfaction, reduced sales, loss of market share, and eventually affects the brand's perception by the farmers.

### 3.2 Bottlenecks affecting maize varietal replacement in SSA

Both supply- and demand-side factors affect the rate of varietal turnover. Fisher et al. (2015) indicated that insufficient availability of seed of recently released varieties affects varietal replacement in Ethiopia, Tanzania, Uganda, and Zambia. Simtowe et al. (2019) attributed the slow adoption of new varieties in Uganda to lack of promotion, limited access to seed of newly commercialized varieties, and high prices of new seed. Smallholder farmers are often risk-averse, have poor access to output markets, poor storage facilities and transport infrastructure, are exposed to counterfeit seed, and have limited access to or inadequate information on the benefits of new varieties (Fisher et al., 2015). Farmers tend to grow the varieties they already know (Rutsaert and Donovan, 2020). The subsistence nature of the many cropping systems in SSA and the possible reluctance of seed companies to invest in replacement of market-dominant old varieties affect varietal turnover (Das et al., 2019).

In the absence of vibrant, private sector driven breeding and dissemination of new varieties, the public sector normally fills the gap. The lack of a profit motive in the public sector to supply seed negatively affects the timely replacement of obsolete varieties. It is cheaper to produce and supply popular, old varieties rather than invest in varietal replacement (Atlin et al., 2017). In a seed industry with limited competition among private seed companies, there is little incentive to replace older, market-dominant varieties with new ones. For example, despite the recent increase in the number of seed companies operating in the ESA (AGRA, 2017), seed markets are still dominated by a few old companies like Kenya Seed Company that controls up to 80% share of the highland market in Kenya (Christinck et al., 2018).

Many emerging seed companies in the ESA have limited infrastructure for direct selling to farmers (Erenstein and Kassie, 2018). The link between farmers and seed companies is mainly through agro-dealers (Rutsaert and Donovan, 2020) who play an important link for varietal turnover, provided seed companies effectively share with agro-dealers technical information and merchandising materials for new varieties, and provide credit mechanisms. Rutsaert and Donovan (2020) found limited interaction between seed companies and agro-dealers in terms of information sharing on new varieties, sales support, and seed promotional materials. Yet about 60% of farmers use agro-dealers as a source of information about seed varieties and agro-dealers were found to be the second main influencers after social networks (farmer-to-farmer) for farmers to switch varieties (Rutsaert and Donovan, 2020). The limited support and interaction between seed companies and agro-dealers is thus a major bottleneck to accelerated varietal replacement in ESA. Seed companies in the ESA need to invest more time and resources to support agro-dealer as drivers of varietal replacement.

Varietal testing and release policies/laws in different ESA countries remain heterogenous and inconsistent (Setimela et al., 2009). The time taken to test and release improved maize varieties varies from two to three years (Table 3). Most countries require both DUS (distinctness, uniformity, and stability) and VCU (value for cultivation and use) data. Yet newer techniques, such as genetic fingerprinting, can effectively replace DUS requirements, as they are more accurate, quicker, and cheaper to implement. VCU data is challenging to generate and should be limited to minimum criteria (e.g.,pest and disease resistance) that can be evaluated in an objective manner, rather than a range of traits that are environment-dependent, labor-intensive, costly, and in most cases fail to account for farmer-preferred traits. In most cases a variety must be tested each time it is introduced in a new country even when developed for, tested and released in similar agro-ecologies in other countries. The recent progress made by regional economic communities in ESA and WA in harmonizing seed trade agreements promises to ease delays in harmonizing varietal release process. However, the harmonized seed policy and regulatory reforms remain to be fully implemented (Table 3). Hence, there is significant scope for improving varietal testing and release policies and procedures across SSA to alleviate one of the major bottlenecks for accelerated varietal turnover.

**Table 3:** Status of seed policy, regulations, and length of maize varietal release process in selected countries in the ESA (in 2020).

	Seed Policy/Law	Ethiopia	Kenya	Malawi	Mozambique	South Africa	Tanzania	Uganda	Zambia	Zimbabwe
Length of variety release process (months)		46	34	24	24	15	31	20	24	18
Status and implementation of national seed policy framework	Seed policy (year)	1993	2010	2018	No seed policy	2012	2013	2018	1999	No seed policy
	Amendment to seed law (year)	2013	2012	2018	No Law	2015	2014	2007	1995	1971
	Seed regulations (year)	2016	2016	2018	2013	2017	2017	2017	2016	2016
	Regional Harmonization status	Under amendment (COMESA)	Harmonized (COMESA)	Under amendment (COMESA & SADC)	Harmonized (SADC)	Not harmonized	Under amendment (SADC)	Harmonized (COMESA)	Pending approval (COMESA)	Harmonized (COMESA)

Based on the information provided by Mabaya et al. (2021).

Reducing the breeding cycle time as well as the time from product development to varietal release, and further from varietal release to varietal deployment increases the ability of breeders and stakeholders to respond rapidly to the climate change-induced challenges and enhances the likelihood that the new products are well-positioned to tackle various abiotic and biotic stresses. Each year of delay in the release/adoption of new varieties with better genetics results in a compounding loss to the farmers of potential yield increment that breeding programs aim to deliver.

Seed companies do encounter financial, product and market risks (see section 4) when launching a new variety in the market. Launching a new product entails considerable up-front investment, increased overhead costs (aside from the investment in the testing and release), and costs related to inventory management, product promotion, etc., while at the same time the small- and medium-sized seed companies often face reduced revenues from earlier released varieties due to market competition and declining seed sales. Similarly, farmers are concerned about the risks they assume when committing to a new or an unfamiliar variety. An extensive on-farm testing system can help mitigate some of these risks, by building confidence in the farmers about product performance.

Maize-based food security in SSA can be significantly improved by delivering to the farmers high-quality seed of recently developed, climate-resilient varieties (Prasanna et al., 2021a). However, quite often farmers are exposed to poor quality seed that forces them to lose trust in seed from the formal seed sector. This negatively impacts varietal turnover. Regulatory agencies need to impose severe penalties to deter the sale of poor-quality seed.

The limited availability of early generation seed (EGS), including breeder, pre-basic and basic seed, is considered as one of the major bottlenecks in ESA (Atilaw et al., 2017). EGS is required for successive scale-up of certified seed. Many seed producers are dependent on data provided by breeding institutions (including data on synchronization and hybrid parent yields) that may have been generated in environments that differ from the company's own seed production environments. In addition, production of EGS requires germplasm knowledge and technical skills, which many of the new entrant small seed companies lack. When EGS is outsourced, demand forecasting becomes a challenge to the producers (Alemu and Bishaw, 2016). Furthermore, low multiplication rates, rejections, losses in the fields, and seed processing constraints affect the availability of EGS. In Ethiopia, Atilaw et al. (2017) found that the number of varieties released in recent years was quite high; however, the number of new varieties with adequate EGS was very low due to the challenges of demand estimation.

### 3.3 Drivers of maize varietal replacement in SSA

Understanding the drivers that stimulate varietal uptake by farmers is critical in formulating effective strategies for varietal replacement (Oladele, 2005). Table 4 summarizes the drivers of varietal replacement. The perception of a new variety by farmers is largely influenced by the firm's marketing strategies. When new varieties are introduced to the market, seed companies need to implement a suite of promotional activities to convince farmers to make replacement purchases. After product launch, awareness can be generated through word-of-mouth from the agro-dealers, and through farmer-to-farmer linkages, i.e., social networks (Foster and Rosenzweig, 2010; Maertens and Barrett, 2012). Demand creation activities by the private sector have leveraged the power of social networks to popularize new varieties. Farmers often rely on such informal communication channels to exchange information about new varieties (Rutsaert and Donovan, 2020). Increased demand for new varieties by individual farmers has been positively correlated with earlier adoption by one or more influential farmers in their community (Foster and Rosenzweig, 2010).

**Table 4:** Factors that drive maize varietal replacement in ESA.

Supply-side factors	Demand-side factors
• Speed breeding programs (e.g., doubled haploidy and marker-assisted breeding to accelerate the rate of genetic gain and product development).	• Multi-channel promotional activities: advertisements, demonstrations, seed fairs, product launches, etc.
• Seed regulatory framework and regional harmonization of varietal testing and release process/laws.	• Farmers' affordability and willingness to purchase seed of new varieties
• Seed producibility* (reduced cost of goods sold)	• Farmers' awareness of and availability of new varieties in agro-dealer shops
• Efficient seed systems (e.g., less cumbersome varietal testing and release laws; existence of an effective seed certification scheme that guarantees quality seed to farmers, etc.)	• Farmers' risk appetite
• Competition in the seed industry for market share	• Perceived potential yield advantage/profitability of the new versus old varieties
• Availability of new, improved varieties with demonstrated tolerance/resistance to key stresses	• Farmers' ability/willingness to invest in other inputs (e.g., irrigation, fertilizer) and good agronomic practices required to make investment in improved seed worthwhile
• Prominent display of seed of new varieties in the shelf space of agro-dealer shops	• Intended grain use from production - subsistence versus commercial
• Effectiveness of the interface between breeding institutions, seed companies, and agro-dealer networks	• Farmer's education level
• Cost of launching a new product in a market vis-a-vis the size of the market or market potential	• Existence of structured output markets
• Quality seed production	• Effective extension program by the company or government
	• Increased per capita food consumption
	• Point of sales technical support/varietal information at agro-dealer level
	• Social networks (farmer-to-farmer)
	• Outbreaks of new devastating pests and diseases

*Seed producibility: For hybrids, the ease with which seed of new varieties can be produced is a function primarily of the female parent seed yield, pollen production capacity of male parent, ease of detasseling the female parent, and the difference in the flowering times of the male and female parents, often termed as “production split” or “nicking”.

Understanding various types of interactions, such as genotype-by-environment, genotype-by-management, genotype-by-environment-by-management, and genotype-by-environment-by-management-by-operating environment, is crucial for stimulating active varietal replacement in different socio-economic and agroecological contexts. Effective product design and positioning rely on a strong understanding of farmers’ management practices and socioeconomic contexts in the target markets. Seed companies and genetics providers need to constantly probe: what (genotype) works, where (environment/management), when (cropping season), and for whom (socioeconomic context)? Robust new varieties need to be evaluated adequately in different environments using farmer-managed conditions to effectively match available products to appropriate markets. Product design (breeding programs) must consider socioeconomic conditions as well as agro-ecological factors.

### 3.4 Potential genetic gain from rapid varietal turnover

Progress in varietal improvement is measured in terms of genetic gain (Tollenaar, 1989), and more recently developed varieties generally confer higher genetic gain (Badu-Apraku et al., 2015). For example, an average gain in grain yield of 13.5 kg ha^−1^ yr^−1^ was reported under drought (Badu-Apraku et al., 2013a), 41 kg ha^−1^ yr^−1^ under Striga (Badu-Apraku et al., 2013b), and 30 kg ha^−1^ yr^−1^ under combined multiple stresses (drought, Striga, and low soil nitrogen) (Badu-Apraku et al., 2015). Between 30 and 60% of farmers' productivity can be traced back to genetic gain (Smith et al., 2015), while the remainder is due to improved agronomy, better markets, and extension systems. Breeding for stress tolerance has resulted in genetic gains under conditions that are similar to farmers' conditions (Weber et al., 2013), and sometimes averted losses after the emergence of new devastating diseases (Singh et al., 2011). Masuka et al. (2017a) found genetic gain within the CIMMYT-derived maize hybrids from 2000 to 2010 to be 109.4, 32.5, 22.7, 20.9, and 141.3 kg ha^−1^ yr^−1^ under optimum, managed drought, random drought, low N, and MSV, respectively. Using data from CIMMYT-derived OPVs bred from 1999 to 2011, Masuka et al. (2017b) further demonstrated genetic gains ranging from 109.9 to 192.9 kg ha^−1^ yr^−1^ in the early-maturing and of 42.3 to 108.7 kg ha^−1^ yr^−1^ in the medium-late maturing varieties. The benefits of these genetic gains can only be realized in the farmers’ fields when PLC management strategies are implemented by seed companies, and the improved genetics is combined with good agronomic management by the farmers.

## 4 Product life cycle management for rapid varietal turnover

**Fig. 1:**
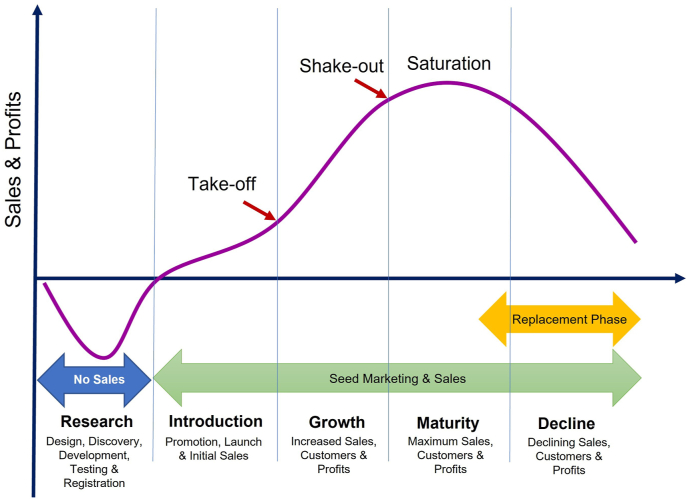
Schematic illustration of a product life cycle. “Take-off” is the point where intensive growth of sales and profits of the introduced variety begin to occur. “Shake-out” stage is when revenue growth, cash flows, and profits start to slow down as the variety approaches maturity.

PLC is the unit sales curve for a variety (from development until its decline and ultimate replacement) and is divided into five phases: development, introduction, growth, maturity, and decline (Fig. 1). PLC management is the effective control of the company's products across their life cycle phases (Stark, 2015); a proactive company begins the process of varietal replacement in the decline phase of the life cycle of an existing product, if not before. Active PLC management is critical if the gains from recent breeding efforts are to be realized and sustained. In Ethiopia, CSA (2010) reported grain yield increment in maize due to active varietal replacement. Synchronizing supply and demand at the beginning and at the end of a variety's life cycle can be challenging and requires effective control of both variety development and its introduction, and ultimately its replacement process. There are limited studies in the seed industry exploring the duration of PLCs and their consequences in SSA. Accurate estimation of the length of PLCs of different varieties in the market requires considerations of only varieties that complete the cycle and those being actively marketed (Magnier et al., 2010). Those that have been removed from the market before completing the cycle for various reasons are not included in the analysis as they distort the PLC duration.

The average duration of varieties in the seed market should be related to the number of new varieties registered each year (MacRobert, 2009). However, in practice, the rate of new variety releases may not be in synchrony with product removal. This is particularly true in most ESA countries, excluding South Africa where nearly 75% of varieties in the market are less than seven years old (MacRobert, 2009). Shorter PLCs should be associated with rapid varietal development and product innovation. PLC management is critical to avoid the pitfalls associated with different stages, and should be an integral part of every seed company's business strategy, rather than approached on an *ad hoc* or reactive basis. Implementation of PLC management requires risk assessment, monitoring of market dynamics, financial investment, and adoption of a contingency strategy.

**Fig. 2:**
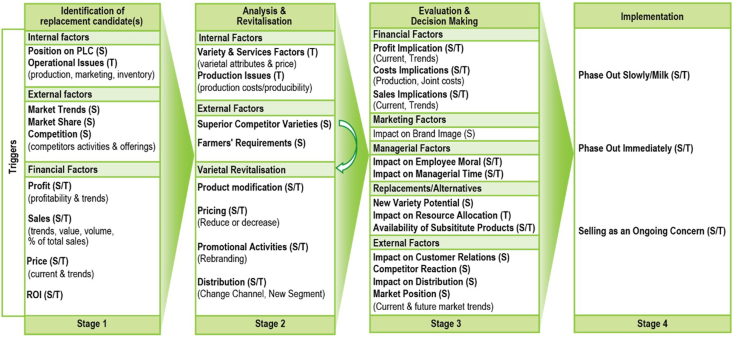
Varietal replacement process, and strategic (S) and tactical (T) variables (modified from Muir and Reynolds, 2011). PLC = Product life cycle; ROI = Return on Investment.

Varietal replacement creates its own set of strategic and management challenges for an organization: should the firm synchronize varietal launch and removal, or sell both the old and the new varieties alongside each other? and how should the firm manage the transition from the old to the newly introduced variety? A robust and responsive varietal replacement process is critical to the success of a newly introduced variety. Product replacement studies (Muir and Reynolds, 2011) noted that replacement is a four-stage process: a) identification of the product to replace; b) analyzing the weaknesses of the target product to replace; c) evaluation and resolution to replace the product; and d) execution of the replacement strategy. While literature shows that the strategic and tactical factors associated with product replacement decision-making differ according to firms’ internal factors, some common strategic and tactical variables correspond to each stage of the product replacement process (Muir and Reynolds, 2011). Fig. 2 outlines different components related to each of the phases of varietal replacement decision-making process, and the strategic and tactical variables associated with each of the stages.

Varietal replacement variables can be strategic (the overarching plan), tactical (the specific steps to make the strategy a reality), or both. While some can be strategic at the organizational level, some could be more tactical in terms of product replacement decision making (Muir and Reynolds, 2011). Financial variables are clearly of strategic concern at the company level (e.g., profit). Yet, within the product replacement context, they act as triggers to, or measures of the viability of potential candidates for replacement. When a firm considers replacing the existing market-dominant variety, both tactical and strategic elements are relevant: the decision to revitalize a variety or implement a replacement plan is strategic; yet the way it is implemented is tactical.

Seed companies use different varietal replacement strategies, considering variables related to timing, production, inventory management, pricing, target segments, farmers’ feedback, and monitoring penetration rates of the new variety. Managers continuously gather market information, update risks, and adapt the strategy. While there is no particular strategy that can be considered as the best, one may classify varietal replacement implementation strategies into two broad types (Billington et al., 1998): primary and contingency. The first is a planned strategy a firm selects at the beginning of the varietal replacement process, depending on the risks of replacing the variety and the market. As more information becomes available about the variety and market, the firm might also adopt a contingency strategy.

Primary replacement approaches can be solo-varietal or dual-varietal rollout. Solo-varietal rollout strategy involves complete liquidation of the old variety inventory before a new variety is introduced. In a dual-variety rollout strategy, firms sell the old and new varieties side-by-side during the new variety introductory phases. The solo-variety rollout strategy has low costs when market conditions turn out as planned. However, this strategy is risky, and it is difficult to synchronize supply-chain operations or sales of the old variety so that it is sold out by the time the new variety is launched. The seed company runs the risk of losing the market share if the old variety is sold out before the new variety is introduced. If the inventory level of the old variety is high at the time the new one is introduced, the company might be forced to sell the old at a discount or incur write-offs resulting in a very expensive rollout strategy.

Smallholder farmers are not homogeneous when it comes to the pace of adoption of new varieties in the market; some adopt quickly while others take time. In such scenarios, the dual-variety rollout strategy is less risky but requires efficiency in coordination and flexibility in the production, delivery, marketing, and pricing of the two products. This strategy runs the risk of confusing farmers due to the side-by-side presence of the two varieties on the market. Four options can be adopted in dual-variety rollout strategy: a) stagger introductions over time in different geographical regions; b) introduce the new variety first in a few targeted market segments; c) dual-pricing strategy – selling the two products at different prices; d) ‘silent’ dual-variety rollout: the new variety is ‘leaked’ deliberately on the market especially if seed of the old variety is in short supply.

**Fig. 3:**
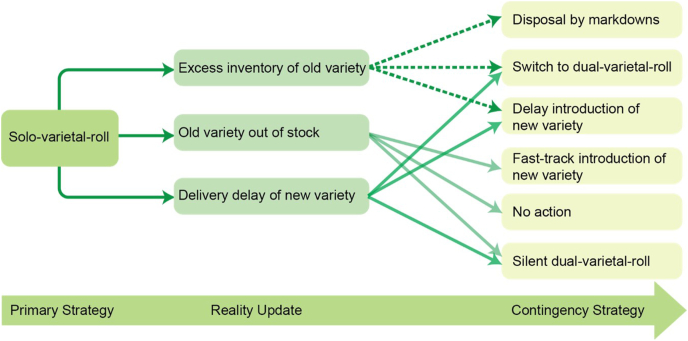
Solo-varietal-rollout strategy (modified from Billington et al., 1998).

When the market conditions shift from the original plan, seed companies may adopt contingency planning (Fig. 3): a) sell the old variety at reduced price to get rid of excess inventory of the old variety: applies when the company selects to pursue solo-variety rollout strategy and the sale of the old variety is unexpectedly slow by the time the new variety is introduced; b) postpone the launch date of the new variety: applies when the company encounters problems with seed production and volumes are low at the time of ramp-up; c) introduce the new variety earlier than originally planned: becomes convenient when the old variety stocks-out prior to the planned launch date; and d) implement two or more dual-variety rollout strategies: happens when the company is faced with an excessive inventory of the old variety or when an insufficient volume of the new is produced. In any of these scenarios, effective varietal replacement requires robust execution of the adopted strategy, including continual monitoring of market dynamics and risks to implement a contingency position if the market dictates.

Successful varietal replacement requires continual monitoring of external factors to identify potential risks and develop and deploy contingency strategies in a timely manner. Risks associated with product replacement include financial, product, and market (Billington et al., 1998). Product (supply) risks relate to the capacity of the company to produce and supply the right quality and quantities of the old and new varieties while managing the product life cycles. Market (demand) risks pertain to the relative demand for the new or old varieties. Financial risks involve the gain or loss to the company because of replacement of the old with the new variety.

### 4.1 Varietal replacement scenarios

Varietal replacement is triggered by different scenarios, which can be influenced by various factors.

#### 4.1.1 Replacement due to external forces

This scenario is a result of outside forces or stimuli that are beyond the company's control. A typical example is the outbreak of a new and devastating disease or pest, causing low sales volume or poor performance of a susceptible variety on the market. If the financial impact is large enough, the seed company must replace the existing variety with an improved one, provided better options are readily available (including varieties resistant to new diseases and pests).

#### 4.1.2 Replacement as a part of product life cycle management policy

Most of the varieties in this group could be marginal products that have reached the decline stage in their PLC. The decision to remove them is triggered by the falling sales volume, profit margins, and increasing negative perceptions from the market. This replacement is characterized by an evaluation of the potential impact of varietal removal in the market, effect on company's market share, reactions of farmers towards removal, and implications on the company's finances. The management also examines the inventory levels of the parental seed to determine the removal strategy.

#### 4.1.3 Replacement of an unsuccessful new variety

When a newly introduced variety falls short of profitability expectations and shows no prospects of improvement, replacement is indeed a must. Recognition of an unsuccessful variety normally comes through poor acceptance by the farmers due to various factors including susceptibility to diseases (e.g., BH543 in Ethiopia, section 4.2). The company typically fails to recover its costs because these varieties are still at the introductory stage. The decision to discontinue the variety is a difficult one for management because it represents a failed investment. Therefore, the evaluation of the causes is thorough, focusing on the market feedback to inform corrective actions and design of future replacement varieties. The effects of the variety's removal on the company's image and relationship with farmers affects how the company's future varieties will be perceived and needs to be handled with care. This entails thorough evaluation of the performance of the future replacement varieties to avoid a cascade of negative perceptions.

#### 4.1.4 Replacement of a market-dominant variety

Replacement of a market-dominant variety is not a trivial task. Despite its ‘weakness’ of being “old genetics”, it may still account for a significant percentage of the company's sales turnover and profitability. Some “old varieties” may also have certain traits desired by farmers and consumers (e.g., SR52 and SC701 in South Africa, H614D in Kenya, and SC513 in Zimbabwe). In addition, the possible market reaction following its removal will still be a grey area. The removal of such varieties is normally preceded by a comprehensive and systematic evaluation process to ascertain whether it is in the best interest of the company and farmers to remove and replace the “cash cow”. The strategy revolves around continuing to ‘‘milk’’ the variety while finding a suitable replacement, which in most cases does not come easily. For example, market-dominant varieties like H614D in Kenya, released in 1986 (Spielman and Smale, 2017) is still being marketed, albeit at a reduced rate (Rutsaert et al., 2019). Research shows that removal of market-dominant products takes several years (Avlonitis et al., 2000). Its reduction in volumes as a phasing out strategy is also sometimes associated with a price increase to optimize the variety's profitability and to use the cash proceeds to fund its replacement.

### 4.2 Maize varietal replacement: some successes in the ESA

**Fig. 4:**
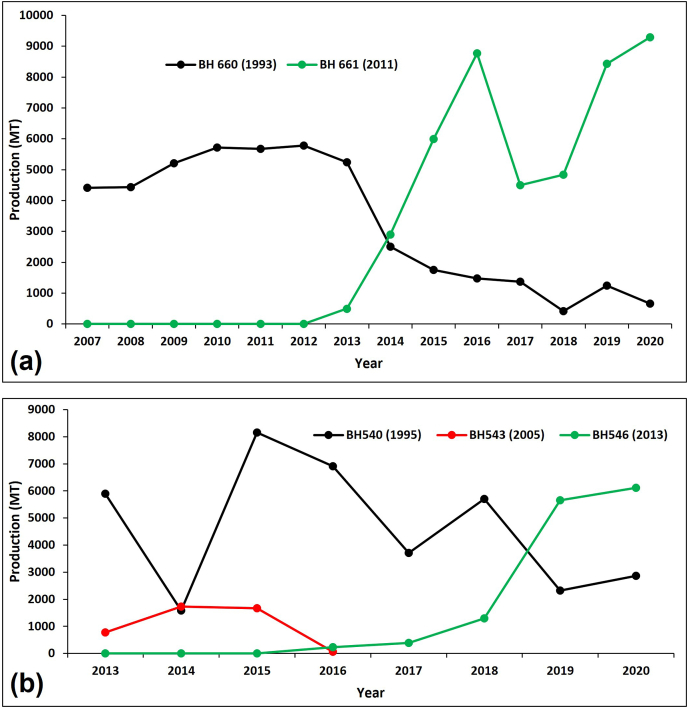
Certified seed production of **(a)** BH660 versus BH661; and **(b)** BH540 and BH543 versus BH546 in Ethiopia. Figures in parenthesis are years of variety official release. Source: Based on Ertiro et al. (2019) and authors' compilation.

Despite the slow varietal turnover in ESA, some successes have been recorded in some countries. For example, variety BH661 bred for mid-altitude Ethiopian markets (Fig. 4a) combines excellent drought tolerance with high yield, standability, and easy seed production (Ertiro et al., 2019). After its release in 2011, farmers quickly adopted the variety, triggering its fast-track production. Farmers were exposed to the variety through a suite of promotional packages, including extensive promotion by extension agents. By 2020, more than 9,000 MT of certified seed was marketed and sold by more than four state-owned seed enterprises in Ethiopia. The new variety is rapidly replacing the previously popular variety BH660 (Fig. 4a), released in 1993 (Abate et al., 2015; Ertiro et al., 2019), representing a scenario outlined in section 4.1 – replacement of a market-dominant variety.

Secondly, a medium-maturing hybrid, BH546, replaced BH540 in 2019 (Fig. 4b). BH540 was released in 1995 and dominated the moist mid-altitude maize hybrid seed market for more than two decades. A decade later, BH543 was released but failed due to susceptibility to diseases like *Turcicum* leaf blight (Fig. 4b), typically representing the case of an unsuccessful new variety (section 4.1). Most seed companies that rely on public germplasm multiplied BH540 as their cash cow in the mid-altitude market until 2019. BH546 was released in 2013 to replace BH540 in the mid-altitude sub-humid market segment. BH546 outperformed both BH540 and BH543 in grain yield by about 28–32% and 27–36%, respectively (Legesse et al., 2012, 2018). BH546 is now very popular among both farmers and seed companies and is replacing BH540 (Fig. 4b). Seed production of BH540 has dropped from about 4,000 in 2018 to 2,870 MT in 2020 while BH546 increased from 800 to more than 6,000 MT by 2020. Several examples of successful varietal replacement exist and remain to be documented in SSA.

## 5 Lessons for future maize varietal turnover in SSA

Organizations’ success and growth stem from proper planning of product portfolios, through new product introduction and/or removal of the old from the market (Cooper et al., 1999). In agriculture, new varietal development research and adoption have received considerable academic and managerial attention. There are limited studies on varietal replacement and PLC management in the seed industry. Studies on product replacement in other industries (which may nonetheless contain valuable and relevant lessons for seed industry stakeholders) indicate that slow product turnover can have a profound effect on brand perception, customer loyalty, productivity, market share, and ultimately profitability of the company. The general perception is that varietal replacement follows declining sales of the current variety on the market. However, effective varietal replacement decisions can be taken when the old variety is still popular. Farmers and seed companies alike benefit from timely replacement of obsolete varieties with new and superior ones: while farmers improve their productivity and resilience to climate-induced stresses and emergence of new biotic threats, seed companies maintain market share and brand relevance.

Farmers may be willing to change varieties, but if seed companies do not expose them to better genetics at affordable cost, they will continue to either recycle the varieties or grow the seed of obsolete but popular varieties on the market. Seed companies have a critical role to play in timely and proactive removal of obsolete varieties from the market. Asymmetric adoption patterns create an opportunity for rapid varietal replacement (Chandrasekaran and Tellis, 2007). Farmers can be categorized into early adopters, late adopters, and laggards. Replacing an old variety with a new variety may be a key strategy to keep the early adopters attracted to the brand. The strategy, therefore, is to identify and target the early adopters with the newest products. Related to the importance of early adopters, is that of opinion leaders (Feick and Price, 1987). Early adopters and opinion leaders tend to influence varietal replacement. Furthermore, certain customers could be “market mavens” – those customers tend to adopt broader products and have market knowledge (Walsh et al., 2004; Harrigan et al., 2021). Market mavens are often the source of information to other customers (Feick and Price, 1987). Literature shows that early adopters, opinion leaders, and market mavens have a great impact on subsequent adoption behavior of other customers (Feick and Price, 1987; Harrigan et al., 2021).

Undoubtedly, the firms’ profitability and market leadership depend on active product replacement or effective product life cycle management (Billington et al., 1998). In other industries, 40% of new products fail on introduction to the market (Ettlie, 1997). The literature on product launch failure in the seed industry is scarce, maybe due to the proprietary nature of the information or lack of diagnostic research on the subject. To minimize the risks of varietal replacement failures, small seed companies with limited resources may benefit from forming cross-functional teams to develop strategies to manage varietal replacement. These cross-functional teams should consist of research, finance, processing, marketing, information systems, sales, and distribution. However, there is no documented process to help the seed company managers to effectively deal with the crucial strategic issues. Successful varietal replacement process requires careful planning, coordination, and execution, including proper management of excess inventory of the old variety, which can potentially delay the introduction of the new variety. Accurate timing is crucial; otherwise, the firm runs the risk of varieties cannibalizing each other, leading to huge inventory and financial loss. Conversely, excessively optimistic sales forecasts can kill the new varietal launch.

The issues related to slow varietal turnover are cross-sectoral and cross-disciplinary. Due to this complexity, these can be resolved only by considering the supply, demand, regulatory environment, and output markets. Enabling policies to simplify and harmonize varietal testing, release and timely replacement are critical. Structured output markets characterized by the presence of overarching supply chain structures that provide coordination and information exchange between seed companies, grain producers, processors, end users, financial institutions, and stable grain prices also stimulate varietal turnover. Governments should prioritize climate-resilient and nutritionally enriched varieties in the procurement and distribution systems, especially through seed subsidy/relief programs. Governmental seed tendering systems in many countries in SSA at present tend to favor low-cost, old, and obsolete OPV seed as more seed can be procured for distribution with limited resources. More often, seed produced by state-owned enterprises is given preference in the tendering process. Such practices introduce unfair competition besides affecting the national maize yields because of the lower performance of older OPVs at the farm level. Therefore, policies should be enacted that promote fair business practices that contribute to accelerated varietal turnover, climate change adaptation and nutritional well-being of the populations.

## 6 Conclusions

This review gives some critical insights into the bottlenecks, drivers, risks, benefits, processes, strategies, scenarios, and examples of varietal replacement for maize in ESA. The quality of seed grown by farmers is one of many important factors that influence the productivity and profitability, besides agronomic, climatic, and social factors. In the absence of active varietal turnover, farmers are indeed missing out on better genetics by continuing to grow old and obsolete varieties, and hence are much more exposed to the risks of climate change and other emerging threats. While it is difficult to be prescriptive on the process to be followed for maize varietal replacement, we strongly suggest that:

• Seed companies should consider proactive varietal replacement as an on-going strategic activity for the benefit of the company as well as for the farmers in SSA who are grappling with various factors affecting crop productivity and resilience to an array of biotic and abiotic stresses.
• Varietal replacement decisions by the seed companies should be data-driven, taking into consideration the availability of extensive on-farm varietal performance data, feedback of the farmers, and consumers.
• Seed companies need to institutionalize the culture of varietal replacement and active PLC management to remain relevant and competitive in the market.
• Slow varietal turnover is affected by complex cross-sectoral and cross-disciplinary issues that require appropriate policy interventions, including streamlining and regional harmonization of varietal testing and release laws; proper enforcement of seed quality regulations; structured output markets; stable grain prices; and prioritization of modern, climate-resilient and nutritionally enriched crop varieties in the procurement systems during government seed subsidy/relief programs.

This review is the first detailed analysis of maize varietal replacement and PLC management in the seed industry in SSA. However, a lot of work needs to be done in this important area. The current state of knowledge on product replacement and PLC management is based on literature from the non-seed industry as empirical studies from the seed industry in SSA are almost non-existent. Therefore, analysis of the dynamic behavior of PLCs in the maize seed industry in SSA requires attention. Further studies on factors that seed companies consider, and their influence on varietal replacement strategies need to be conducted. Farmers' and agro-dealers’ reactions to varietal removal also await future exploration. The effect of shortening the PLCs and expansion of varieties on inventory costs must be explored. The impact of slow varietal turnover on brand perception, firm profitability, farmer productivity, and consequently food security is another attractive area for future research. Finally, it is hoped that this review might stimulate research interests in varietal replacement and firms’ PLC management practices in the seed industry in SSA.

## Declaration of competing interest

The authors declare the following financial interests/personal relationships which may be considered as potential competing interests: The authors of this review are employed by CIMMYT, an international public agricultural research organization that receives funding from public and private sources to support its maize and wheat breeding programs. Although the conclusions drawn by the authors of this review may support the interests of their employer as well as the funding agencies, we have striven to maintain objectivity.

## Footnotes

1 The term “variety” in this manuscript refers to both maize hybrids and open-pollinated varieties (OPVs) released and cultivated by the farmers.
